# Can Tuberculous Matter Taken as Food Excite Tuberculous Disease?

**Published:** 1876-08

**Authors:** 


Can Tuberculous Matter Taken as Food Excite Tuberculous Disease?—Prof. Gurlack (*Edinburg Medical Journal*, October, 1875,) gives an answer to this inquiry based upon experiments. He fed animals one or two doses of tuberculous matter by the stomach. The effects were observed, and if the animal did not die it was killed after some weeks and a post mortem examination made. As conclusion he says:

(1) There is a specific virulent material in tubercle, and many of the symptoms of tubercular disease are due to the absorption of this virus.

(2) This virus exists in tubercle in all its stages, but apparently in greater intensity in cheesy masses. It is found in recently formed tubercle, and in miliary tubercle.

(3) The infection begins first in the mucous membrane of the mouth, and if the tubercular matter be in contact a sufficient length of time with the mucous membrane of the alimentary canal, it may communicate the disease to the whole lymphatic system.

(4) While tubercular disease has special characters in different animals, all tubercular matter, when introduced into the alimentary canal from one species to another, is more or less virulent.

(5) The tubercular matter of birds, especially that of the common hen, is very virulent, and is identical in its action with that of mammalia.

(6) The fibrous tubercle of horses without a trace of cheesy formation, is just as infectious as the miliary tubercle of cattle.

(7) The flesh of tuberculous animals is also infectious, though in a much less degree than tubercle itself.

(8) Tubercular material cooked for a quarter of an hour is still infectious, though in a less degree than that not cooked.

(9) The effects of poisoning by tubercular matter taken into the alimentary canal, are irritation of the mucous membrane both of the alimentary and respiratory tracts, enlargement and tenderness of the lymphatic glands, enlargement of the bronchial glands, and the formation of tubercle in the lungs and other organs.—*Detroit Review of Medicine.*

